# Combinatorial treatment of acute myocardial infarction using stem cells and their derived exosomes resulted in improved heart performance

**DOI:** 10.1186/s13287-019-1353-3

**Published:** 2019-10-10

**Authors:** Peisen Huang, Li Wang, Qing Li, Jun Xu, Junyan Xu, Yuyan Xiong, Guihao Chen, Haiyan Qian, Chen Jin, Yuan Yu, Jiandong Liu, Li Qian, Yuejin Yang

**Affiliations:** 1 State Key Laboratory of Cardiovascular Disease, Fuwai Hospital, National Center for Cardiovascular Diseases, Chinese Academy of Medical Sciences and Peking Union Medical College, No.167 Bei Li Shi Road, Xicheng District, Beijing, 100037 People’s Republic of China; 2 McAllister Heart Institute, University of North Carolina at Chapel Hill, Chapel Hill, NC 27599 USA; 3 Department of Pathology and Laboratory Medicine, University of North Carolina at Chapel Hill, Chapel Hill, NC 27599 USA; 4 Department of Cardiology, the First Affiliated Hospital, Sun Yat-Sen University, Guangzhou, 510080 People’s Republic of China; 5 Chapel Hill, USA

**Keywords:** Exosomes, MSCs, SDF-1, Myocardial infarction

## Abstract

**Background:**

Bone marrow mesenchymal stem cells (MSCs) are among the most common cell types to be used and studied for cardiac regeneration. Low survival rate and difficult retention of delivered MSCs in infarcted heart remain as major challenges in the field. Co-delivery of stem cell-derived exosomes (Exo) is expected to improve the recruitment and survival of transplanted MSCs.

**Methods:**

Exo was isolated from MSCs and delivered to an acute myocardial infarction (AMI) rat heart through intramyocardial injection with or without intravenous infusion of atrovastatin-pretreated MSCs on day 1, day 3, or day 7 after infarction. Echocardiography was performed to evaluate cardiac function. Histological analysis and ELISA test were performed to assess angiogenesis, SDF-1, and inflammatory factor expression in the infarct border zone. The anti-apoptosis effect of Exo on MSCs was evaluated using flow cytometry and Hoechst 33342 staining assay.

**Results:**

We found that intramyocardial delivery of Exo followed by MSC transplantation (in brief, Exo+MSC treatment) into MI hearts further improved cardiac function, reduced infarct size, and increased neovascularization when compared to controls treated with Exo or MSCs alone. Of note, comparing the three co-transplanting groups, intramyocardially injecting Exo 30 min after AMI combined with MSCs transplantation at day 3 after AMI achieved the highest improvement in heart function. The observed enhanced heart function is likely due to an improved microenvironment via Exo injection, which is exemplified as reduced inflammatory responses and better MSC recruitment and retention. Furthermore, we demonstrated that pre-transplantation injection of Exo enhanced survival of MSCs and reduced their apoptosis both in vitro and in vivo.

**Conclusions:**

Combinatorial delivery of exosomes and stem cells in a sequential manner effectively reduces scar size and restores heart function after AMI. This approach may represent as an alternative promising strategy for stem cell-based heart repair and therapy.

## Introduction

Ischemic heart disease (IHD) is the leading cause of mortality around the world, accounting for 16.6% of all death in 2016 [1]. Stem cell therapy has been recognized as a promising approach for the treatment of IHD via replenishing lost cells after acute myocardial infarction (AMI). Accumulating preclinical studies have shown that transplantation of various types of stem cells, such as bone marrow-derived mononuclear cells (BMMNCs), mesenchymal stem cells (MSCs), circulating progenitor cells (CPCs), embryonic stem cells (ESCs), and induced pluripotent stem cells (iPSCs) and their derivatives improved cardiac function after AMI [2, 3]. However, limited studies achieved significant improvement in cardiac contractile function and scar reduction after AMI due to poor engraftment and survival of transplanted cells in the ischemic heart [2–4].

The interaction between stromal cell-derived factor 1 (SDF-1) and its receptor CXC chemokine receptor 4 (CXCR4) has been demonstrated to play a vital role in the recruitment of stem cells to the ischemic myocardium [5–8]. It has been shown that the expression of SDF-1 in ischemic cardiac tissues is transiently elevated and peaked at 1 to 3 days after ischemic insult and decreases to the background level in the following few days [8–10]. Consequently, transplantation of stem cells during early days after AMI achieved higher cell recruitment rate [10]. However, acute inflammatory reaction and highly oxidative stress at the same stage of elevated SDF-1 expression is detrimental for the survival of transplanted cells [11]. Enhancing SDF-1 expression while dampening inflammatory level may help to promote the cell engraftment and survival, thus to improve the subsequent therapeutic efficiency [12, 13].

Exosomes are extracellular, membrane-bound vesicles with a diameter of 30–150 nm and contain a wide range of functional proteins, mRNAs, and microRNAs (miRNAs) [14, 15]. They originate intracellularly and are actively secreted by most cell types. Exosomes play an important role in intercellular and tissue-level communication by transferring their cargo of bioactive molecules between cells. Recent studies suggested that MSC-derived exosomes (Exo) could serve as potential cell-free therapy for cardiac repair with the ability to augment cell survival, proliferation, neovascularization, and ameliorate inflammatory reaction in infarcted heart [16–20]. It remains unclear whether Exo could synergize with stem cells to further improve efficacy of cardiac stem cell transplantation therapy.

In this study, we investigated the therapeutic effects of sequential delivery of Exo and MSCs on rat AMI hearts and explored the optimal time window for the delivery. Exo treatment followed by MSC delivery at early stage after AMI further improved cardiac function and stimulated angiogenesis compared to MSC delivery alone. In particular, MSC injection 3 days after Exo led to reduced inflammatory levels and increased expression of SDF-1, which in turn promoted MSC retention and survival, thereby leading to improved heart performance. Our data thus suggests that sequential delivery of Exo and MSCs may represent as an alternative promising strategy for stem cell-based heart repair and therapy.

## Methods

### Animal use

All animals were obtained from the Experimental Animal Center of Fuwai Hospital (Beijing, China). All experiments conformed to the National Institute of Health Guide for Care and Use of Laboratory Animals and were approved by the Experimental Animals Ethics Committee of Fuwai Hospital.

### Bone marrow MSC isolation and cell culture

MSCs were obtained from male Sprague-Dawley (SD) rats (60–80 g) as previously described [21]. In brief, bone marrow was flushed with medium from the femur and tibia and seeded in culture dishes containing Iscove’s Modified Dulbecco’s Medium (IMDM, Invitrogen) with 10% fetal bovine serum (FBS, Gibco) and penicillin (100 U/mL)/streptomycin (100 μg/mL) (HyClone). After 24 h of culturing at 37 °C in a humidified incubator with 5% CO_2_, the medium was changed to remove the non-adherent cells. When they grew to about 90% confluence, MSCs were harvested and passaged at a ratio of 1:3. MSCs at passage 3 were identified by flow cytometry with antibodies against the cell surface markers of CD45 (Ebioscience), CD29 (Ebioscience), CD90 (Ebioscience), and CD11 (BD Biosciences). Passage 3–4 MSCs were used in this study.

### Exosome extraction and identification

Exosomes were isolated by differential centrifugation as previously described [22]. In brief, MSCs were cultured in exosome-free FBS containing IMDM for 48 h, which was previously centrifuged at 120,000*g* for 18 h to remove pre-existing bovine-derived exosomes. Then, the conditioned supernatant was collected and centrifuged. Cells and debris were eliminated by centrifugation at 300*g* for 10 min and 2000*g* for 20 min respectively, and macrovesicles were removed at 13500*g* for 30 min. The supernatant continues to be ultracentrifuged at 120,000*g* for 70 min to obtain the crude exosome pellet. The pellet was subsequently washed with phosphate-buffered saline (PBS, pH 7.4) followed by repeat ultracentrifugation for 70 min at the same speed. The exosome pellet was resuspended in appropriate volume of PBS and stored at − 80 °C for the use in the experiments. The entire operation was performed in sterile condition.

The protein concentrations of Exo were measured by microBCA protein assay kit (Thermo Scientific). Then, the shape and size of exosomes were identified by transmission electron microscope (TEM, HITACHI, H-600IV) and Nanoparticle Tracking Analysis (NTA, Malvern Instruments, UK). In addition, the Exo were identified by using Western blot with antibodies against CD81 (Cell Signaling Technology), CD63 (Cell Signaling Technology), Alix (Cell Signaling Technology), and TSG101 (Cell Signaling Technology), which were previously described as specific exosome markers [14, 23].

### Internalization of PKH26-labeled exosomes into MSCs

To determine whether exosomes can be efficiently internalized by MSCs both in vitro and in vivo, purified exosomes were labeled with fluorescent dye PKH26 using Red Fluorescent Cell Linker Kit (Sigma-Aldrich) according to the manufacturer’s protocol and washed in PBS followed by 2 times of ultracentrifugation to remove the extra dye. The PKH26 pre-labeled MSC-derived exosomes (Exo-PKH26, 2 μg/ml) were added into MSCs in culture and incubated for 12 h; then, the cells were washed with PBS, fixed with 4% paraformaldehyde, and stained with fluorescent antibody against smooth muscle α-actin (α-SMA) and DAPI at room temperature. Uptake of labeled exosomes by cells was determined using confocal microscopy. For in vivo experiments, 10 μg pre-labeled exosomes were injected to the border zone of infarcted heart 30 min after infarction. Distribution of Exo-PKH26 in infarcted heart was monitored on day 1, day 3, and day 7 after injection by immunofluorescence, and the photographs were taken under confocal microscopy (LSM 780, Zeiss).

### Rat AMI model induction

All surgeries and subsequent analyses were performed in a blinded fashion for intervention. Animals were randomized into different treatment groups. Female Sprague-Dawley rats (200–220 g weight) were anesthetized by intraperitoneal injection of 100 mg/kg ketamine combined with 10 mg/kg xylazine and ventilated via tracheal intubations connected to a rodent ventilator. AMI surgery was performed by ligation of left anterior descending coronary artery with a 6-0 silk suture as described previously [21, 24]. AMI induction was verified by loss of color in the region below the ligation area.

### Pretreatment of MSCs and implantation of exosome and MSCs

Our previous study reported that pretreating MSCs with 1 μM atorvastatin (ATV, one of the widely used lipid-lowering drugs for patients with coronary heart disease) for 12 h enhanced the migration of MSCs to the infarct site by increasing the expression of CXCR4 on the surface of MSCs. This improved the homing and survival of MSCs infused through tail vein and improved cardiac function after AMI [21]. Therefore, it is feasible to transplant MSCs through systemic infusion, which will be more convenient for future clinical use. Thus, in this study, all the MSCs were pretreated with 1 μM ATV for 12 h and injected through tail vein.

Rats were randomized into Sham; AMI (PBS alone); Exo (exosomes treatment alone); MSC^d1^, MSC^d3^, and MSC^d7^ (MSCs transplantation alone at d1, d3, d7 post AMI respectively), and Exo+MSC^d1^, Exo+MSC^d3^, and Exo+MSC^d7^ groups (Exo combined with MSCs transplantation at d1, d3, d7 post AMI respectively). The Sham group underwent the same surgical procedures except for the permanent ligation step. Exo (10 μg, in 100 μl PBS) or PBS (AMI group) were injected at three sites around the border zone of infarcted heart 30 min after left anterior descending coronary artery ligation using a 31-gauge Hamilton syringe. For stem cells transplantation groups, 2 × 10^6^ MSCs were injected into the Exo or PBS delivered rats through the tail vein at 1 day, 3 days, or 7 days post AMI. Heart function, infarct area, and angiogenesis were evaluated at 5 weeks post infarction.

### Cardiac function

Rats were anesthetized for analysis with a mixture of 1.5% isoflurane and oxygen (0.8 L/min). Transthoracic two-dimensional M-mode echocardiography was performed to evaluate the cardiac function at baseline (3 days post MI) and endpoint (5 weeks post MI) using the Vevo 2000 system (VisualSonics). M-mode were used to measure left ventricular wall thickness and left ventricular inner diameter in systole and diastole. The mean value of three measurements was determined for each sample. The variation of the left ventricular ejection fraction (LVEF), fractional shortening (FS), left ventricular end-diastolic volume (LVEDV), and left ventricular end-systolic volume (LVESV) between baseline and endpoint were calculated. All procedures and analysis were conducted by a researcher who was blinded to treatments.

### Histological analysis

Five weeks after AMI, animals were euthanized to remove the hearts. Both Masson trichrome staining (Sigma) and Sirius Red staining were used for determining infarct size and fibrosis, and hematoxylin-eosin (HE) staining was performed to evaluate inflammatory cell infiltration. In brief, the hearts were simply trimmed to eliminate the upper part of ligation after immersion in 4% formaldehyde for 48 h. The remainder of the heart was cut at 1 mm below the ligation point perpendicular to the axis of the left anterior descending coronary artery and processed with paraffin embedding. To elucidate the severity of myocardial infarction, the slices with 4 μm of paraffin tissue were stained with Masson trichrome to measure the average ratio of the fibrotic area to the entire LV cross-sectional area (infarct area/LV%), and Sirius Red staining was used to calculate the average ratio of collagen area to entire LV area (collagen area/LV %), with the use of Image J software (Version 1.51j8, NIH) [25–27].

### Immunohistochemical analysis

To assess angiogenesis and SDF-1 expression in the infarct border zone, the heart tissue sections were deparaffinized, rehydrated, permeabilized, and then stained with conjugated antibodies against CD31 (Abcam), α-SMA (Abcam), or SDF-1 (Santa Cruz). Then, the sections were incubated with respective secondary antibodies conjugated with Alexa fluor 488 (Invitrogen) or color reaction with the DAB kit. The samples were analyzed using a confocal microscope (LSM 780, Zeiss). Positively stained cells were counted in three sections per heart, five high-power fields (HPFs) per section. Vascular density was quantified as the number of CD31+ cells per HPF. Arteriole density was quantified as the number of α-SMA+ cells per HPF. SDF-1 expression in peri-infarcted heart tissue at 1 day, 3 days, and 7 days after AMI was also quantitatively evaluated by rat SDF-1 ELISA kit (Elabscience, E-EL-R0922c) according to the manufacturer’s instructions.

### Apoptosis assay

MSCs were plated on 6-well plates (1 × 10^5^ cells/well) and pretreated by PBS or 2 μg/ml of Exo for 12 h, then changed to DMEM without glucose and FBS and induced apoptosis by H/SD condition. Cells were put into a sealed GENbox hypoxic chamber fitted with an AnaeroPack (Mitsubishi Gas Chemical Company, Japan) to scavenge the free oxygen at 37 °C for 10 h. The oxygen concentration was maintained below 0.1% as indicated by an Anaer indicator (Bio-Merieux, Marcy I’ Etoile, France). The anti-apoptosis effect of exosomes on MSCs was evaluated using flow cytometry and Hoechst 33342 staining assay.

### Statistical analysis

Data were given as mean ± standard error of mean (SEM). Continuous variables were compared by the Student *t* test. Multiple comparisons were performed by one-way ANOVA followed by Tukey’s test, two-way ANOVA, or repeated-measure two-way ANOVA followed by Tukey post hoc test. Data was analyzed with SPSS 22 (IBM) or GraphPad Prism 7 for Windows, version 7.01 (GraphPad Software, Inc.). Graphs were assembled in GraphPad Prism 7. *P* < 0.05 was considered to indicate a significant difference.

## Results

### Characterization of exosomes derived from MSCs

**Fig. 1:**
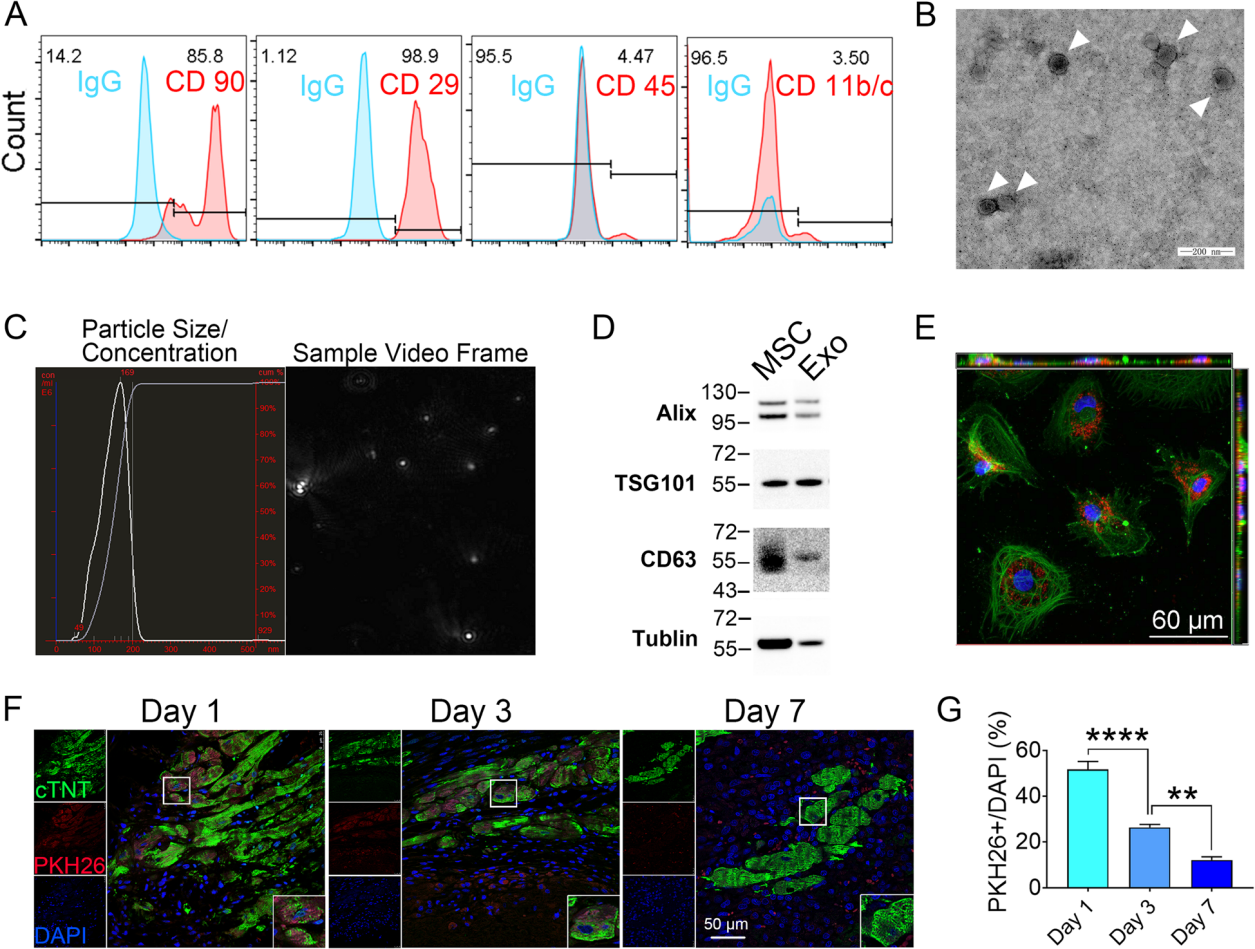
Characterization and functional validation of exosomes derived from MSCs (Exo). **a** Phenotypic analysis of cell surface antigens of MSCs by flow cytometry. **b** Cup-shaped morphology of purified Exo assessed by transmission electron microscopy. Scale bar = 200 nm. **c** The particle size, particle concentration, and video frame of Exo analyzed by nanoparticle tracking analysis. **d** Representative images of Western blot showing the exosomal protein markers. **e** Representative confocal images showing that red fluorescence dye PKH26-labeled Exo (Exo-PKH26) were endocytosed by MSCs after12-h incubation. Scale bar = 60 μm. **f** Distribution of Exo-PKH26 in the infarcted heart on day 1, day 3, and day 7 post injection. Scale bar = 50 μm. **g** Quantification of PKH26+ cells in **f** (*n* = 5). (A-G) *n* = 5. All data are mean ± SEM. Statistical analysis was performed with one-way ANOVA followed by Tukey’s test. **p < 0.01, ****p < 0.0001

To obtain sufficient and high-quality exosomes, we enriched exosomes from MSCs derived from rat bone marrow. MSCs at passages 3–4 were typically adherent spindle-shaped cells and were recognized as CD90+ CD29+ CD45− CD11− cells analyzed by flow cytometry (Fig. 1a). Exosomes isolated from MSCs were spheroids or typical cup-shaped vesicles with an average size of ~ 100 nm as visualized using TEM and NTA (Fig. 1b, c). The isolated exosomes contained high level of exosome-specific proteins such as Alix, TSG101, and CD63 (Fig. 1d). PKH26 pre-labeled Exo can be efficiently internalized by co-cultured cells in 12 h and localized in cytoplasm, suggesting they were intact and functional (Fig. 1e). Injection of Exo-PKH26 into injured hearts showed peaked retention of Exo at 24 h after MI, and gradually dropped from day 3 to day 7 (Fig. 1f, g).

### Combined Exo and MSC transplantation improved post AMI cardiac function

**Fig. 2:**
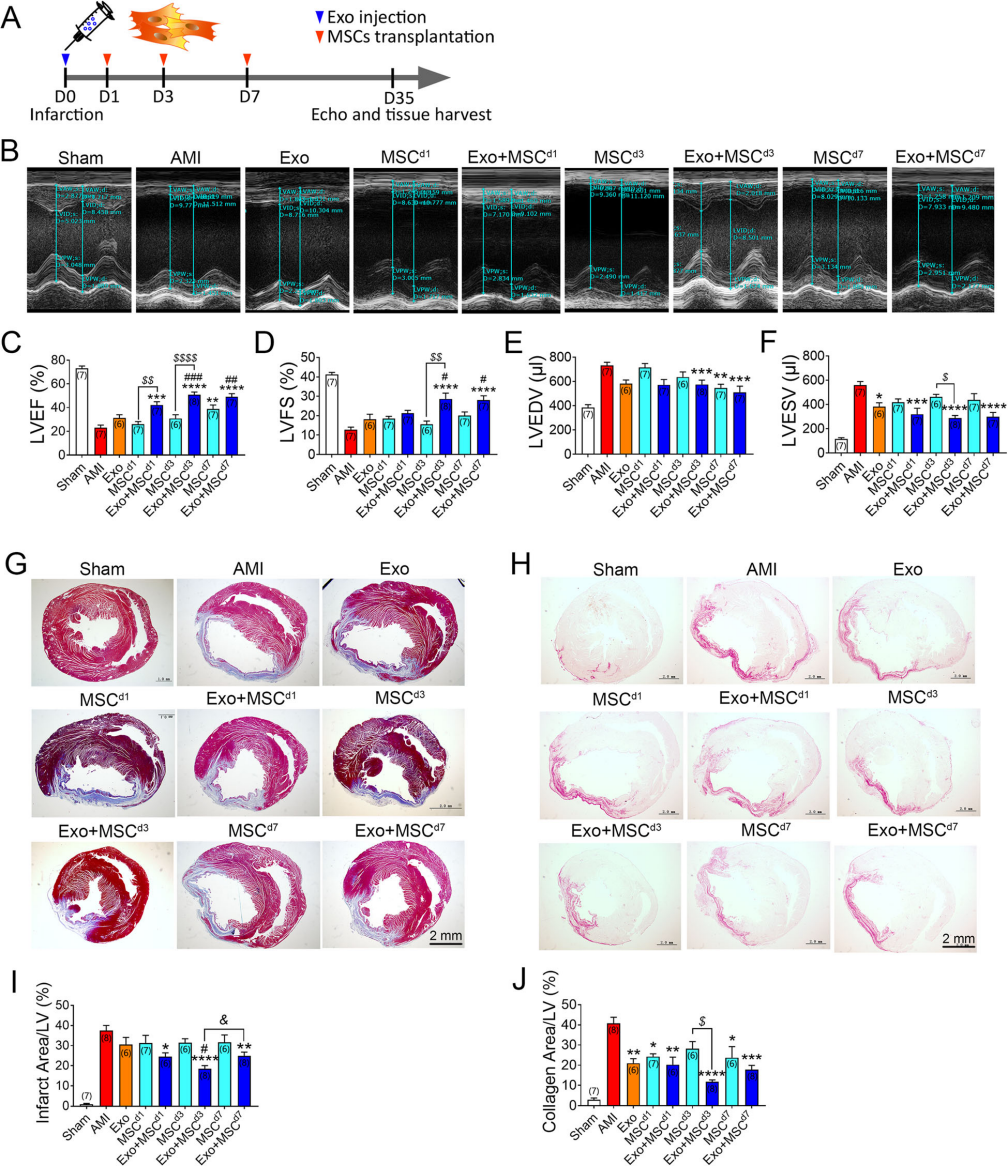
Exo and MSCs synergistically improved cardiac function and ameliorated fibrosis after AMI. **a** Schematic of the sequential delivering of Exo followed by MSCs into the infarcted hearts experiment. **b** Representative echocardiogram of rat hearts in different groups 5 weeks post AMI. **c**, **f** Significantly enhanced left ventricular ejection fraction (LVEF), fractional shortening (LVFS), left ventricular end-diastolic volume (LVEDV), and left ventricular end-systolic volume (LVESV) in rats co-transplanted with Exo and MSCs compared with AMI, Exo, and MSCs alone groups (*n* = 6–8 for each group). **g** Representative transverse heart sections analyzed with Masson trichrome staining at 5 weeks after AMI. Red, myocardium; blue, scarred fibrosis. Scale bar = 2 mm. **h** Representative Sirius Red staining images for collagen analyze in each group. Scale bar = 2 mm. **i**, **j** Quantitative data for the LV fibrotic area and the quantitative data of Masson trichrome staining (**i**) and Sirius Red staining (**j**) (*n* = 6–8 for each group). All data are mean ± SEM. Statistical analysis was performed with two-way ANOVA followed by Tukey post hoc test. *p < 0.05, **p < 0.01, ***p < 0.001, ****p < 0.0001 vs. AMI group; ^#^*p* < 0.05, ^##^*p* < 0.01, ^###^*p* < 0.01 vs. Exo group; ^*$*^*p* < 0.05, ^*$$*^*p* < 0.01, ^*$$$$*^*p* < 0.0001 vs. MSC group; ^*&*^*p* < 0.05 vs. Exo + MSC^d7^ group

To determine the potential effect of combinatory delivery of Exo and MSCs in vivo, Exo or PBS was intramyocardially injected into the border zone of infarcted rat hearts 30 min after injury. In consideration of different retention rate of Exo and the possible functional outcome it may cause, we designed experiments where MSCs were injected into the rats through the tail vein at 1 day, 3 days, or 7 days post AMI-Exo treatment (Fig. 2a). While AMI weakened the contractility of left ventricle (LV) anterior walls compared to Sham operation, Exo and MSC combined delivery significantly mitigated AMI-induced LV dilation on day 35 (Fig. 2b). In the AMI control group, a significant decrease in LVEF and LVFS were observed, suggesting compromised cardiac function. Delivery of Exo alone gave rise to increase in LVEF and LVFS and significant decrease in LVESV but not in LVEDV. Delivery of MSCs alone at 7 days after MI showed significant improvement in LV contractility and function. We then explored the optimal time window for combined Exo and MSC delivery (Fig. 2c–f). In combination of Exo administered on the day of MI (d0), no further improvement of heart function was found if MSCs were injected on day 1 (Exo + MSC^d1^) compared to groups treated with either Exo or MSCs alone. In contrast, delivery of MSCs on day 3 (Exo + MSC^d3^) and day 7 (Exo + MSC^d7^) resulted in a further improvement of both LVEF and LVFS over other groups (Fig. 2c, d). MSCs injected 3 days after Exo delivery (Exo + MSC^d3^) achieved the best improvement of cardiac function among all three combination groups, although no statistically significant relationship was found (Fig. 2c–f).

Because Exo and MSCs combinatorial treatment groups demonstrated preserved cardiac function, we were interested to evaluate the infarct size and collagen area. At 5 weeks post infarction, all of the Exo and MSC alone groups showed no significant decrease in infarct size when compared with AMI control group. In contrast, rat hearts receiving Exo and MSC combinatorial treatment had significantly smaller infarct size. Importantly, the reduction in infarct size was more prominent in Exo + MSC^d3^ group when compared with that in Exo alone and Exo + MSC^d7^ group (Fig. 2g, i). Consistent with the echocardiogram results, collagen area decreased significantly in all of the therapeutic groups, but the infarcted hearts treated with MSCs at day 3 followed by Exo delivery demonstrated further decreased collagen area compared with those in the MSC^d3^ group (Fig. 2h, j). Of note, Exo + MSC^d3^ group achieved the lowest infarct area and collagen positive area among all three combination groups (Fig. 2i, j). Together, these results indicated that Exo and MSC combinatorial treatment after AMI resulted in an enhanced therapeutic effect, and 3 days after MI-Exo treatment might be the best time point for sequential stem cells delivery.

### Exo and MSC combinatorial therapy augmented neovascularization and myocyte survival after AMI

**Fig. 3:**
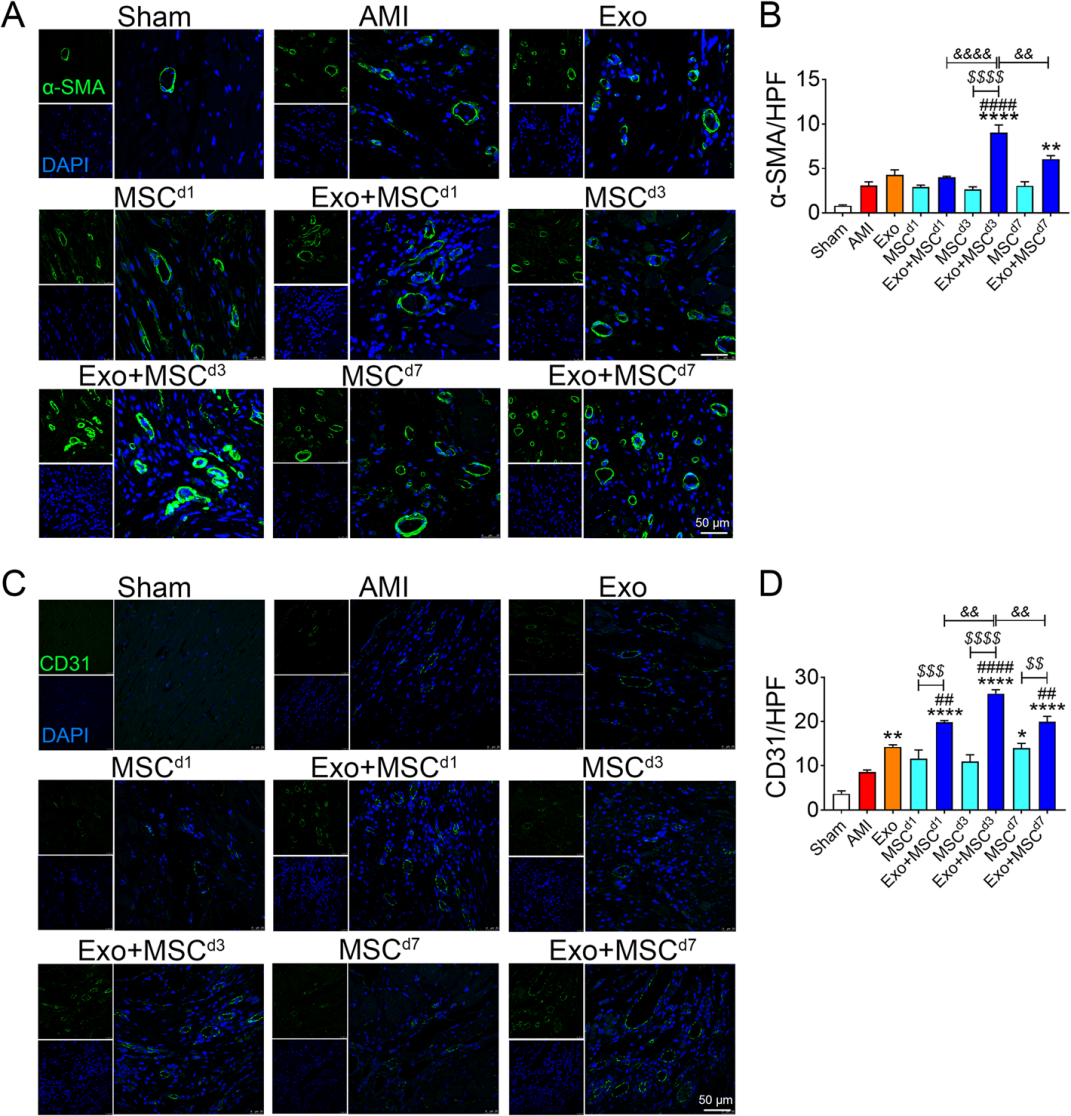
Exo and MSCs combined therapy promoted angiogenesis in the infarcted hearts. **a** Arterioles at the border zone on 5 weeks after AMI were identified by staining with α-SMA (green) and nuclei (blue). **b** Quantification of α-SMA+ cells in A (*n* = 5). **c** Capillary at the border zone on 5 weeks after AMI was identified by staining with CD31 (green) and nuclei (blue). **d** Quantification of CD31+ cells in **c** (*n* = 5). All data are mean ± SEM. Statistical analysis was performed with two-way ANOVA followed by Tukey post hoc test. *p < 0.05, **p < 0.01, ****p < 0.0001 vs. AMI group; ^##^*p* < 0.01, ^####^*p* < 0.0001 vs. Exo group; ^*$$*^*p* < 0.01, ^*$$$*^*p* < 0.001, ^*$$$$*^*p* < 0.0001 vs. MSC group; ^*&&*^*p* < 0.01, ^*&&&&*^*p* < 0.0001 vs. Exo + MSC^d1^ and Exo + MSC^d7^ groups. Scale bar = 50 μm

To investigate whether Exo and MSC combinatorial therapy induces morphometric changes in the infarcted heart, we assessed vascular density in peri-infarct myocardium by quantification of α-SMA staining on the smooth muscle lining arterioles, and CD 31 staining on the luminal side of vascular endothelial cells (Fig. 3). Exo and MSC alone groups failed to increase arteriolar density when compared with AMI group. In contrast, arteriolar density was significantly increased by the Exo and MSC combinatorial treatment. Comparing the three combinatory treatment groups, Exo + MSC^d3^ showed the highest increase in microvascular density compared with Exo + MSC^d1^ and Exo + MSC^d7^. This increase in microvascular density was observed in both arteriolar and capillary vessels (Fig. 3a–d). Increased capillary density was also found in Exo and MSC^d7^ alone groups while there was no significant change in MSC^d1^ and MSC^d3^ groups (Fig. 3c, d).

### Exo adapted cardiac environment through reducing inflammation and enhancing SDF-1 expression to improve MSCs recruitment and retention

**Fig. 4:**
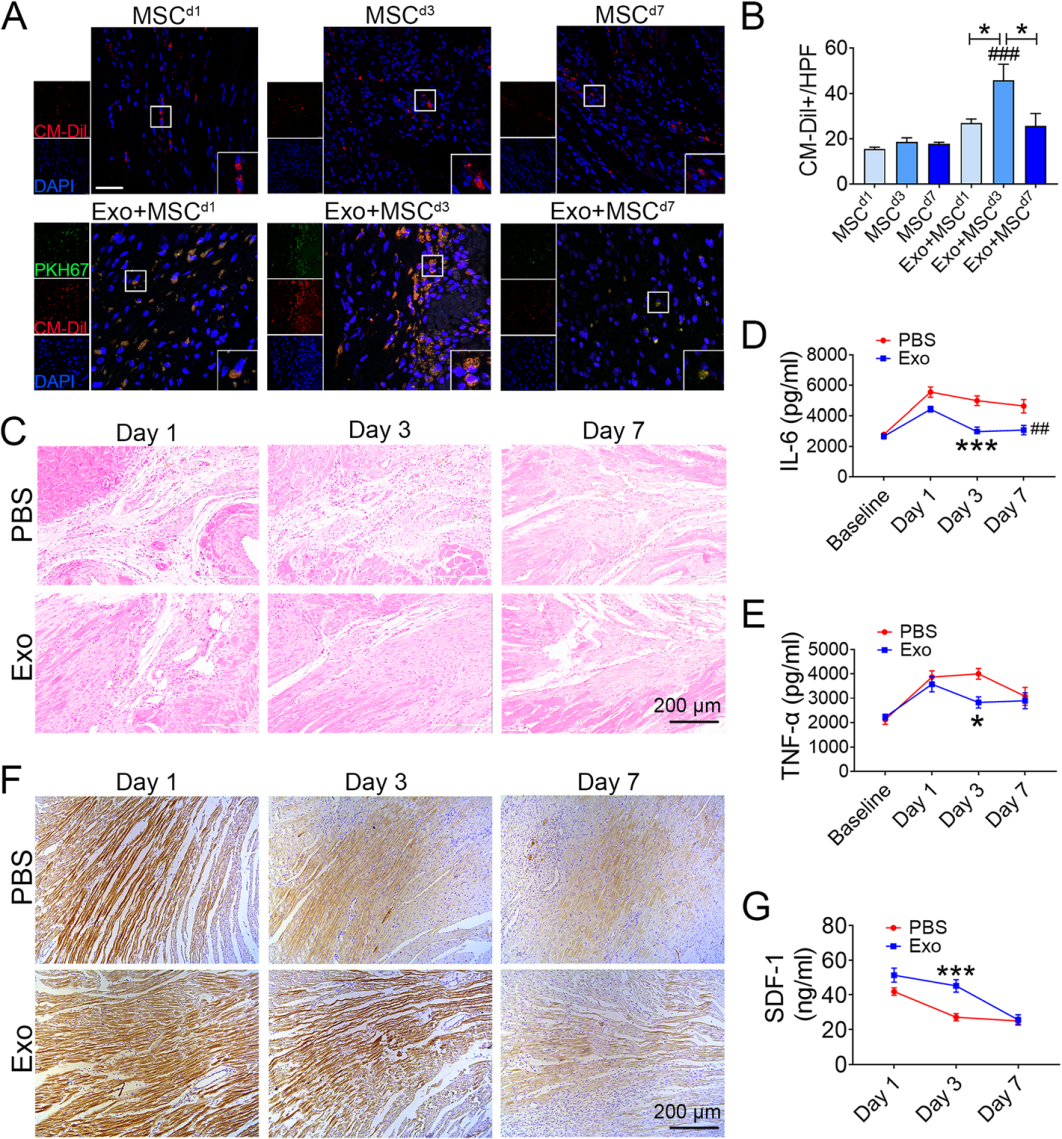
Exo pretreatment provided a relatively low inflammatory reaction and relatively high level of SDF-1 micro-environment in infarcted hearts for transplanted MSCs to survive. **a** Distribution of MSCs pre-labeled with CM-Dil (red) in the infarcted hearts on 5 weeks post AMI in the groups with and without PKH67 pre-labeled Exo (green) injection. Scale bar = 100 μm. **b** Quantification of CM-Dil+ cells in A (*n* = 5). **c** Representative HE staining images at the border zone 5 weeks after AMI. Scale bar = 200 μm. **d**, **e** Quantification of IL-6 and TNF-α expression level in the infarct border zone tissue of rat hearts using ELISA method (*n* = 5). **f** Representative immunohistochemical staining images of SDF-1 in PBS and Exo groups in 1 day, 3 days, and 7 days post AMI (× 200). Scale bar = 200 μm. **g** ELISA assessment of SDF-1 expression during the time course. All data are mean ± SEM. Statistical analysis was performed with one-way ANOVA followed by Tukey’s test. *p < 0.05, **p < 0.01, ***p < 0.001, ****p < 0.0001; ^*##*^*p* < 0.01, ^*###*^*p* < 0.001

To better elucidate the mechanisms underlying the hemodynamic and morphometric improvements observed by the Exo and MSCs combined therapy, we evaluated the distribution and recruitment/retention of transplanted MSCs in infarcted myocardium through fluorescent dye tracing. We transplanted CM-Dil-labeled MSCs to the MI rat hearts at day 1, day 3, or day 7 with or without additional treatment with PKH67 pre-labeled Exo. Heart sections in these animals at 5 weeks post MI were analyzed and we found that significant more CM-Dil+ cells were found in Exo and MSC combinatorial treatment groups compared to MSC alone groups. In addition, CM-Dil+ cells were significantly increased in Exo + MSC^d3^ hearts compared with Exo + MSC^d1^ (1.7-fold) and Exo + MSC^d7^ hearts (1.8-fold, Fig. 4a, b). Collectively, these results indicated that retention of transplanted MSCs in the myocardium could be achieved when Exo was delivered on the day of MI injury followed by MSC delivery at day 3 after Exo injection.

Severe inflammation in myocardial microenvironment post MI is detrimental to the survival of transplanted cells. Since the interaction between SDF-1and CXCR4 plays a vital role in the engraftment of MSCs in the injured myocardium [6, 9], we therefore determined the inflammation level and SDF-1 expression in infarcted hearts to further investigate potential mechanisms underlying the enhanced migration/retention observed in the day 3 MSC combinatorial therapy group. H&E staining demonstrated that massive inflammatory cell infiltration occurred in infarcted hearts during the first week post AMI and that Exo delivery significantly reduced immune cell infiltration at day 3 and day 7 post MI compared to the PBS group (Fig. 4c). Furthermore, ELISA test showed that cytokines such as IL-6 and TNF-α in peri-infarcted heart tissue were decreased significantly compared to the PBS control group on 3 days after Exo delivery (Fig. 4d, e). Thus, the delivered Exo played a potent regulatory role in reducing inflammation in the ischemic myocardium post MI.

Consistent with previous report [8–10], both immunohistochemistry (Fig. 4f) and ELISA (Fig. 4g) results showed that SDF-1 expression in peri-infarcted heart tissue of PBS control group peaked at day 1 post AMI and rapid declined within the first week. In contrast, SDF-1 maintained a relatively high level of expression in Exo transplanted hearts on day 3 post AMI and dropped to a similar level on day 7 as PBS group. Therefore, administration of Exo offered longer time window of maintained upregulation of SDF-1 expression post AMI. Taken together, intramyocardial injection of Exo led to a lower inflammation level and relatively high SDF-1 expression in peri-infarcted myocardium on day 3 after AMI, which probably contributed to the higher recruitment and retention rate of MSCs in the Exo + MSC^d3^ combinatorial therapy group.

### Exo protects MSC against H/SD and reduced apoptosis both in vitro and in vivo

**Fig. 5:**
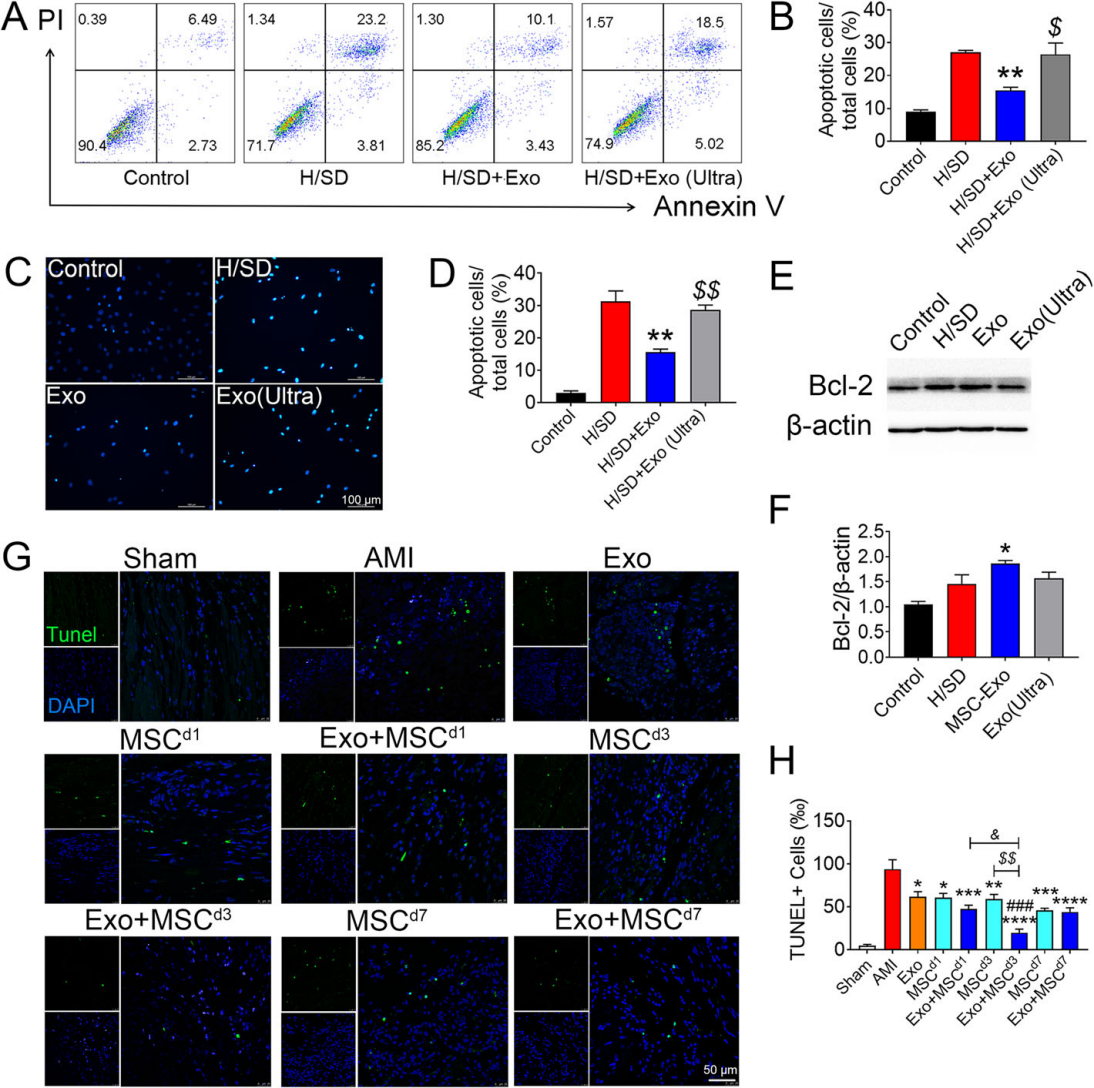
Exo enhanced the survival and decreased the apoptosis of MSCs under hypoxia and serum deprivation (H/SD) conditions. **a**–**d** Flow cytometry assay and Hoechst 33342 nucleic acid stain for apoptosis of MSCs after treated with PBS, Exo, or Exo (Ultra) and exposed to H/SD. **a** Scatter diagram of apoptosis in MSCs treated with Exo and negative control. **b** Histogram of apoptosis events in different groups (*n* = 3). **c** Representative images of Hoechst 33342 nucleic acid stain. Scale bar = 100 μm. **d** Quantification of apoptosis cells in each group (*n* = 3). **e** Western blot for Bcl-2 protein in MSCs treated with Exo or Exo (Ultra) and exposed to H/SD condition. **f** Quantification of Bcl-2 in E (*n* = 3). **g** TUNEL staining at the border zone on 5 weeks post AMI with TUNEL (green) and nuclei (blue). **h** Quantification of TUNEL+ cells in G (*n* = 5). Apoptosis rate was quantified as the percentage of cells that were positive for TUNEL staining. All data are mean ± SEM. Statistical analysis was performed with one-way ANOVA followed by Tukey’s test or two-way ANOVA followed by Tukey post hoc test. *p < 0.05, **p < 0.01, ***p < 0.001, ****p < 0.0001 vs. H/SD or AMI group; ^###^*p* < 0.001 vs. Exo group; ^*$*^*p* < 0.05, ^*$$*^*p* < 0.01 vs. H/SD + Exo or MSC group; ^*&*^*p* < 0.05 vs. Exo + MSC^d1^ group. Scale bar = 50 μm

To determine the effect of Exo on MSC survival, MSCs were treated with Exo for 12 h and then subjected to H/SD in vitro. Flow analyses showed that the H/SD + Exo group demonstrated a significant reduction in apoptosis compared to the H/SD and H/SD + Exo (Ultra) groups, in which exosomes were ruptured by ultrasonic (Fig. 5a, b). Similar result was observed using Hoechst 33342 staining assay (Fig. 5c, d). Interestingly, Exo treatment enhanced the expression of anti-apoptotic protein Bcl-2 in MSCs under H/SD (Fig. 5e, f), which may protect cells from cell death. These results indicated that Exo significantly improved the survival of the MSCs under hypoxic stress.

To further explore the pro-survival effect of Exo and MSC combined therapy in vivo, TUNEL staining was performed in the treated hearts on 5 weeks post MI. The number of apoptotic cells was significantly decreased in the myocardial boarder zone treated with exosomes or MSCs compared to that treated with PBS. Among the three combination therapy groups, only the Exo + MSC^d3^ group was found to statistically further reduce the number of TUNEL+ cells compared with Exo and MSCs alone groups. Importantly, the apoptotic cells also significantly decreased in day 3 combination group when compared with day 1 combination group (Fig. 5g, h). Taken together, these results indicated that pre-treatment with Exo can enhance the ability of MSCs to survive under the H/SD condition in vitro and the ischemic environment in vivo, which may in turn contribute to the improved cardiac function post AMI.

## Discussion

**Fig. 6:**
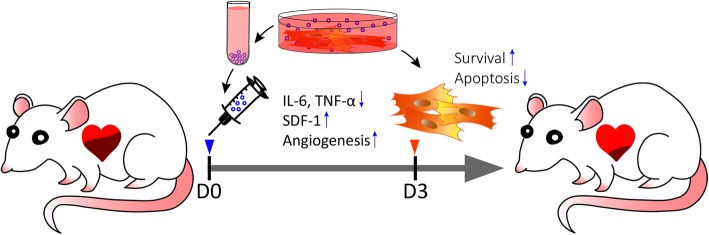
Effects and mechanisms of MSC-derived exosomes and MSC combinatorial therapy on acute myocardial infarction. Delivery of Exo into infarcted hearts 30 min post AMI can significantly reduce inflammatory factors such as IL-6 and TNF-α, increase SDF-1 expression and angiogenesis, and promote cell survival in the stressed ischemic microenvironment at day 3 post AMI. These mechanisms ultimately lead to significant augmentation of cardiac function and reducing scar size of AMI rat hearts after Exo and stem cells combinatorial therapy

Despite the promise shown by various types of stem cells for restoration of cardiac function after AMI, the low recruitment and poor survival of transplanted cells have been a major issue [2, 3, 7]. Delivery of cell-free components, such as exosomes derived from stem cells, has been demonstrated to enhance myocardial viability and reduce or slow down the adverse remodeling of pathological hearts [14, 20]. Therefore, we reasoned that administration of MSC-derived exosomes would modulate the ischemic milieu to facilitate transplantation of stem cells and thus could achieve better cardiac repair/regeneration. This study showed a further improved therapeutic efficacy of sequential delivery of MSCs following Exo injection in a rat AMI model and revealed multifaceted mechanisms that enhanced recruitment and survival of co-transplanted MSCs. Of note, we further demonstrated that 3 days after Exo delivery into infarcted hearts was the optimal time point when the microenvironment is less inflammatory and tolerable for the stem cells to be retained in the ischemic hearts (Fig. 6).

Recent studies have suggested that MSC-derived exosomes carry a wide range of functional proteins, mRNAs, and miRNAs which could serve as a potential cell-free therapeutic for cardiac repair [17, 18, 28]. However, exosomes therapy hardly achieved the goal of myocardial regeneration and/or sustained functional restoration because they lack the ability to promote cardiomyocyte proliferation and regeneration [19, 29]. In addition, the exosome therapy has a short effective lifetime and mild efficacy due to limited retention in the myocardium [19, 29, 30]. In this study, we injected exosomes 30 min after MI and then cells at intervals of 1, 3, and 7 days post MI. Such kind of combined treatment of exosomes with stem cells may be a more viable therapeutic approach for clinical cardiac regeneration. Although a 30-min interval for door to balloon time is too short for clinical application, which is a limitation of this study, this scheme provides reference for the design of future large animal experiments and clinical trials. To our knowledge, this is the first known report of a combinatorial therapy using both stem cell-derived exosome and stem cells in the ischemic myocardium.

One of the main barriers limiting the effectiveness of stem cell transplantation is the stress ischemic microenvironment in which high level of inflammation would damage most of the transplanted cells. To avoid the adverse inflammation during the first few days post AMI, most studies transplanted stem cells 1 to 2 weeks after AMI [3, 31]. However, emerging evidence and our results suggested that the expression of SDF-1, which plays a vital role on MSC migration and survival, increased rapidly within 1 to 3 days post AMI and subsequently decreased to the background level in the following week [8–10] (Fig. 4f, g). These dynamic changes of inflammation and SDF-1 expression make it challenging to identify an optimal intervention time of stem cells and lead to a low recruitment and survival of transplanted cells [11]. In this study, we nstead pre-delivered Exo 3 days before MSC transplantation to alleviate heart injury. This approach created a less inflammatory microenvironment where inflammation was downregulated while SDF-1 remained at relatively high level. This strategy of combinatorial therapy demonstrated higher recruitment and retention rate of transplanted cells as well as superior recovery of heart function.

Exosomes affect stem cell functions in multiple ways. Zhang et al. [32] preconditioned cardiac progenitor cells (CPCs) with MSC-derived exosomes and then transplanted the pretreated CSCs into a rat MI model. They found that the pretreatment of CSCs with Exo had a superior effectiveness in decreasing cardiac fibrosis and increasing survival and capillary density, which finally improved cardiac function. Ong et al. [33] co-delivered exosomes derived from endothelial cells (EC-Exo) with CPCs into MI mice and demonstrated that CPCs are capable of uptaking EC-Exo in 12 h after injection, which could confer increased tolerance to the co-transplanted CPCs at day 7. In our study, we found that prior treatment with Exo rendered MSCs increased tolerance to hypoxia stress both in vitro and in vivo, indicating a consistent positive interaction between stem cell-derived exosomes and stem cells.

In recent years, various novel strategies have been designed and studied to enhance stem cell-based therapies including cell pretreatment, genetic modification, biomaterial approach, and combination cell therapy [20, 34]. We presented here sequential delivery of stem cell-derived exosomes followed by stem cell further improved heart function. These advances demonstrate the promising potential of stem cell therapy, while the goal of full cardiac recovery and regeneration has yet to be realized in either preclinical or clinical researches. Continuing efforts to discover novel multifaceted cell-based therapeutic approaches, such as incorporation of stem cells and exosomes into biomaterials and loading exosomes or stem cells with cell fate reprogramming factors, could accelerate our progress to achieve this goal.

## Conclusions

Combinatorial delivery of exosomes and stem cells in a sequential manner effectively reduces scar size and restores heart function after AMI. Delivery of Exo into infarcted hearts 30 min post AMI can significantly reduce inflammatory factors such as IL-6 and TNF-α, increase SDF-1 expression and angiogenesis, and promote stem cell survival in the stressed ischemic microenvironment at day 3 post AMI. This approach may represent as an alternative promising strategy for stem cell-based heart repair and therapy (Fig. 6).

## Data Availability

All data generated or analyzed during this study are included in this published article.

## Abbreviations

AMI: Acute myocardial infarction
CPCs: Cardiac progenitor cells
CXCR4: CXC chemokine receptor 4
EC: Endothelial cell
ESCs: Embryonic stem cells
Exo: Exosomes derived from MSCs
FBS: Fetal bovine serum
FS: Fractional shortening
H/SD: Hypoxia and serum deprivation
IL-6: Interleukin 6
iPSCs: Induced pluripotent stem cells
LVEDV: Left ventricular end-diastolic volume
LVEF: Left ventricular ejection fraction
LVESV: Left ventricular end-systolic volume
MSCs: Mesenchymal stem cells
PBS: Phosphate-buffered saline
SDF-1: Stromal cell-derived factor 1
TEM: Transmission electron microscopy
TNF-α: Tumor necrosis factor-α
α-SMA: Smooth muscle α-actin
